# 

**Published:** 1893-01

**Authors:** 


### INDEX

TO THE

### HOMŒOPATHIC PHYSICIAN.

### VOLUME XIII, 1893.

PAGE

Abortion, Hemorrhage following. J. A. Tomhagen, M. D., . . . . . . . 246
Abortion. See "Miscarriage,"
Acne, A Case of. D. C. McLaren, M.D., 92
Aconite-nap.$^{50m}$, . . . . . . . . . . . 52
Aconite, . . . . . . . . . . . . . . 50, 287
Actea-racemosa, . . . . . 436, 438, 468, 474
Adams, E. T., M. D. Can Scabies be Cured with the Simillimum? . . 144
Fibroid Tumor of the Uterus, . . 241
Additional Clinical Cases. B. Fincke, M. D., . . . . . . . . . . . . . . 103
Additional Report of Board of Censors, 17
Address to Delinquent Subscribers. Editorial, . . . . . . . . . . . . 561
Adeps, . . . . . . . . . . . . 442, 468, 469
Advice Wanted. W. E. Everly, . . . 118
Æsculus-hip., . . . . . . . . . . 52, 292, 387
Agaric-musc., 50, 52, 287, 292, 385, 390, 438, 442, 469, 489, 491, 531
Ailanthus-gland., . . . . 51, 293, 383, 388
Alaskana, or Alaska. By Prof. Bushrod W. James, A. M., M. D. Review of, . . . . . . . . . . . . 60
Albuminuria. W. L. Reed, M. D., . . 69
Aletris, . . . . . . . 292, 377, 390, 468, 531
Allan, A. G., M. D. Glaucoma, . . . . 3
Some Clinical Cases, . . . . . . . 151
Removal of, . . . . . . . . . . . 600
Allen, J. H., M. D. Erysipelas, . . . 72
Hydro-salpingitis. A Case Cured with Gelseminum, . . . . . . . 244
Two Successful Homœopathic Surgical Operations, . . . . . . 14
Allen, T. F., M. D. Profuse Nocturnal Enuresis, . . . . . . . . . . . . 431
Alumina, . . . . . . . . . . . . . . 468
American Institute of Homœopathy, The. Pemberton Dudley, M. D., 397
Transactions of, . . . . . . . . . 480
Wm. E. Leonard, M. D., . . . . . 555
American Text-Book of the Diseases of Children, An, . . . . . . . . 255
American Text-Book of Surgery for Practitioners and Students. By Dr. Wm. Keen and Dr. J. Wm. White. Review of, . . . . . 63
Ammon-carb., . . . . . . . . . . . . 50, 527
Announcement. E. B. Treat. Note, . 127
Annual Reunion of the Alumni Association of the Hahnemann Medical College, Philadelphia. Note, 256
Antimony, . . . . . . . . . . . . 378, 532
Apis. An Involuntary Proving. Julia C. Jump, M. D., . . . . . . . . 396
Apis, . . . . . . . 290, 292, 432, 469, 474, 493
Apium-virus, . . . . . 292, 377, 383, 442
Apocynum-cann, . 52, 290, 293, 390, 437, 531
Apoplexy, . . . . . . . . . . . . . 525
Appendicitis. James Lilienthal, M. D. 458
Aranea, . . . . . . . . . . . . . 53, 438, 469
Argentium Nitricum. A Case Cured with. W. P. Wesselhœft, M. D., 110
Argent-nit., . . . . . . . . . . . . 397, 531
Arnica in Whooping Cough, . . . . . 169
Arnica, . . . . . . . . . . . . . 51, 169, 527
Arsenicum in Ovaritis. . . . . . . 165, 169
Arsenicum, 53, 287, 293, 377, 383, 385, 388, 390, 432, 436, 437, 442, 469, 527, 531
Arum-trif., 51, 291, 293, 378, 385, 390, 391, 432, 438, 489, 491, 527, 532
Asclepias-tub., . . . . . . . . . . . . 287
Asiatic Cholera, Another Remedy for. A. K. Crawford, M. D., . . . . . 505
Asterias-rub., . . . . . . . . . . . . . 387

PAGE

Atrophine-sulphate, . . . . . . . . . 278
Aurum-mur.$^{10m}$, . . . . . . . . . . . 53
Auto-Isopathy, A Case of. A. McNeil, M. D., . . . . . . . . . . . . . . 372

Ball, G. R., M. D. Is Camphor the Genius Epidemicus of the Present Cholera Invasion? . . . . . 283
Banerjee, D.N., M.D. Calcutta Homœopathic Charitable Dispensary, . 59
Clinical Cases, . . . . . . . . . . 155
A Case of Typhlitis, . . . . . . . 155
A Case of Eczema, . . . . . . . . 156
Cases of Hernia, . . . . . . . . . 157
Baptisia-tinct., . . . . . . . . 293, 378, 527
Baryta-carb., . . . . . . . . . . . . 432
Baylies, B. Le Baron, M. D. Typhoid Pneumonia, . . . . . . . . . . . 81
Beals, Dr. Herbert, removal, . . . . . 304
Beaman, Chas. P., removed, . . . . . 447
Belladonna as a Prophylactic. C. F. Nichols, M. D., . . . . . . . . . . 347
Belladonna Case, A. Why Not Mercurius? W. E. Ledyard, M. D., . . 164
Belladonna, 51, 53, 291, 293, 378, 388, 391, 432, 438, 469, 491, 494
Bell, James B., M. D. The Resignation of Dr. Bell, . . . . . . . . . . . 510
Benzoic-acid, . . . . . . . . . . . . . 293
Betts, B. Frank, M. D. Introductory to the 46th Annual Course of Instruction in Hahnemann Medical College of Philadelphia, . . 563
Boger, Cyrus M., M. D. Repertory of the Symptoms of the Ovaries, . . 494
British Medicinal Plants. Alfred Heath, M. D., . . . . . . . . . . . 394
Book Notices, 59, 251, 299, 349, 445, 478, 511, 560, 559
Book on the Physician Himself, and Things that Concern his Reputation and Success. By D. W. Cathell, M. D. Review of, . . . 302
Borax, . . . . . . . . . . . . . . 293, 532
Bromine, . . . . . . . . . . . . . 293, 432
Bryonia, . . . . . . . . . . . . . . . 438
Bufo, . . . . . . . . . . . . . . . . . 432
Bureau of Clinical Medicine, . . . . . 69
Bureau of Obstetrics, . . . . . . . . . 234
Bursa-pastoris, . 288, 293, 383, 385, 389, 391, 433, 438, 442, 491, 527, 532

Cadmium, . . . . . . . . . . . . . . . 490
Calcarea-carb., The Intermittent of. A. McNeil, M. D., . . . . . . . . 345
Calc-carb., . . . . . . . . . . . . . . 494
Calcutta Homœopathic Charitable Dispensary. D. N. Banerjee, . 59
Calendula-off., . . . . 291, 294, 337, 378, 383
Camphor the Genius Epidemicus of the Present Cholera Invasion? Is. G. R. Ball, M. D., . . . . 283
Camphor, . . . . . . . . . . . . . . 386, 494
Cancer-astacus, . . . . . . . . . . . . 378
Cannabis-sat., . . . . . . . . . . . . 378
Can Scabies be Cured with the Simillimum. E. T. Adams, M. D., . . 144
Capsicum, . . . . . . . . . 78, 294, 433, 469
Carbolic Acid, Accidental Proving of. J. C. Fahnstock, M. D., . . . . . 554
Carb-veg., . . . . . . . . . . . . . 470, 490
Carbuncle. J. N. Lowe, M. D., . . . 23
Cases Cured. By Dr. Dahlke, . . . . 122
Cases from Practice, Dr. Hesse. By A. McNeil, M. D., . . . . . . . . . . 503

PAGE
Cases Cured by Dr. Wassel, Keil. A. McNeil, M. D., . . . . . . . . . . 504
Case, Erastus E. Clinical Cases, . . . 76
Case, Dr. E. E. Complexion and Constitution, . . . . . . . . . . . . . 462
Cases Cured with High and Highest Potencies, . . . . . . . . . . . 283
Caulophyllum, . . . . . . . . . . . . 433
Causticum, . . . . . . . 290, 294, 378, 469, 491
Censors, Additional Report of Board of, 17
Cham., . . . . . . . . . . . . . . . . . 526
Chapman, S. E., M. D., Homœopathic Aggravation, . . . . . . . . . . . 427
Chapter of Cholera for Lay Readers. History and Symptoms; Prevention of the Disease. By Walter Voight, M. D. A Review of, . . 480
Chelidonium. A Case of Eczema Cured by B. N. Banerjee, M. D., . . . . 156
Chelidonum-maj., . . . . . . . . . 289, 439
Chicago Baptist Hospital. Note, . . . 400
Chicago Hotel Rates. Note, . . . . . 304
Childhood. George W. Winterburn, M. D. Review of, . . 61, 128, 304, 351
Childhood's Logic. Note, . . . . . . . 63
Cholera; Another Remedy for, . . . 505
Cholera; Its Prevention and Treatment. By Elmer Lee, M. D. Review of, . . . . . . . . . . . 349
Cholera for Lay Readers; Chapter of. Review of, . . . . . . . . . . . . 480
Cholera; Therapeutics of. By P. C. Majumdar, M. D. Review of . . 600
Cichor-intyb., . . . . . . . . . . . . 294
Cimicifugo-rac., . . . . . . . . 288, 491, 528
Cina, . . . . . . . . . . . . . . 378, 494, 528
Cinnabar, . . . . . . . . . . . . . . . 490
Circular, . . . . . . . . . . . . . . . 56
Clark, Geo. H., M. D. Sycotic Dysmenorrhœa, . . . . . . . . . . . 94
Clematis, . . . . . . . . . . . . . . . 490
Clinical Cases, Some. A.G. Allan, M.D., 151
B. N. Banerjee, M. D., . . . . . . 155
Erastus E. Case, M. D., . . . . . . 76
B. Fincke, M. D., . . . . . . . . . . 98
Jennie Medley, M. D., . . . . . . 112
W. L. Morgan, M. D., . . . . . . . . 141
G. Pompili, M. D., . . . . . . . . . 197
With Remarks. Edward Mahony, M. D., . . . . . . . . . . . . . . 172
Clinical Contribution. T. D. Stow, M. D., . . . . . . . . . . . . . 217
Clinical Medicine. J. A. Tomhagen, M. D., . . . . . . . . . . . . . . 219
Bureau of, . . . . . . . . . . . . . 69
Clinical Notes. Wm. Steinrauf, M. D., 348
Cocaine, Purity of. Note, . . . . . . 512
Cochlearia-a., . . . . . . . 384, 436, 439, 470
Coffea, . . . . . . . . . . . . . . . . 526
Collinsonia, . . . . . . . . . . . . . 442, 470
Comparison of the Ammoniac-salts with Kali and Natrum-salts, . . 279
Comparison of the Throat Symptoms of Lachesis and Lycopodium, A. E. V. Ross, M. D., . . . . . . . . . 259
Compendium of Materia Medica, Therapeutics, and Repertory of the Digestive System, A. By Arkell Roger McMichael. Review of, . . . . . . . . . . . . . 299
Competitive Examination, A, . . . . 255
Complexion and Constitution. Dr. E. E. Case, . . . . . . . . . . . 462
Conium, . . . . . . . . . . . . . . . . 528
Consumption, Diseases of Digestive Organs as a Primary Cause of, . 130
Copaiba, . . . . . . . . . . . . . . . 489
Cornus-florida, . . . . 53, 288, 291, 379, 532
Correction, . . . . . . . . . . . . . . 64
Correction, A. Prof. Dr. G. Jaeger, . . 45
"Corrections" of Prof. Dr. G. Jaeger, from the Hahnemannian Standpoint, The. B. Fincke, M. D., . 119
Crawford, A. K., M. D. Another Remedy for Asiatic Cholera, . . . . 505

PAGE
Croton-tig., . . . . . . . . . . . . . . 435
Crutcher, Howard, M. D. An Illustration, . . . . . . . . . . . . . 347
The Question of Repetition, . . . 396
Cuprum Poisoning, A Case of. John M. E. Ward, M. D., . . . . . . . 280
Cuprum-acet., 53, 294, 379, 388, 391, 433, 436, 439, 470, 532

Dahlke, Dr. Cases Cured, . . . . . . 122
Davis, F. S., M. D. Tumor of the Neck treated by the use of the Knife followed by Death, . . . . . . . 2
Delay in Issuing this Number, The. Editorial. Walter M. James, M. D., . . . . . . . . . . . . . . 1
Delayed August Number, The, . . . . 322
Dever, I., M. D. Siftings Saved, . . . 548
Cases Cured by Psorinum, . . . . 88
Diagnosis, Special, and Homœopathic Treatment. Review of, . . . . . 253
Diet for the Sick. By Miss E. Hibbard. Review of, . . . . . . . . . . . 301
Diet for Wasting Diseases, The, . . . . 63
Digestive Organs, Diseases of as the Primary Cause of Consumption and of many other Diseases. W. L. Morgan, M. D., . . . . . . 130
Digit-purp., . . . 50, 389, 433, 470, 490, 528
Diphtheria, Lycopodium in, . . . . . 166
Diphtheria, Repertory of. Wm. Jefferson Guernsey, M. D., . . . . . . 222
Diseases of the Digestive Organs as the Primary Causes of Consumption and of many other Diseases. W. L. Morgan, M. D., . . . . . . 130
Diseases of Inebriety from Alcohol, Opium, and other Narcotic Drugs, The. Review of, . . . . 303
Diseases of the Lungs, Heart, and Kidneys. By N. S. Davis, M. D. Review of, . . . . . . . . . . . 251
Diseases of the Nose and Throat. By Horace F. Ivins, M. D. Review of, . . . . . . . . . . . . . . . 446
Dolichos-prur., . . . . . . . . . 294, 526, 532
Doryphora, . . . . . . . . . . . . . . 532
Dracontium, . . . . . . . . . . . . . . 439
Dudley Pemberton, M. D. The American Institute of Homœopathy, . 397
Dudley Pemberton, M. D., Secy. A World's Congress of Homœopathy, . . . . . . . . . . . . . . 54
World's Congress Transactions, . 510
During the Cold Weather. Note, . . 127
Dysmenorrhœa, . . . . . . . . . . . 209
Sycotic. Geo. H. Clark, M. D., . . 94

Editorials, . . 1, 65, 129, 195, 257, 305, 353, 401, 449, 481, 513, 561
Editorial in the July Number, The. Edward Payson Small, M. D., . 511
Electro-Homœopathy. Wm. Steinrauf, M. D., . . . . . . . . . . . 399
Emmons, J., M. D. A Few More Cases Cured with the High and Highest Potencies, . . . . . . . . 283
Enuresis, Profuse Nocturnal. T. F. Allen, M. D., . . . . . . . . . . 431
Era Key to the U. S. P., 1893. By Haynes & Co. Review of, . . . 511
Erecthites, . . . . . . . . . . . . . . 433
Erigeron, . . . . . . . . . . . . . . . 492
Erysipelas. J. H. Allen, M. D., . . . 72
Essentials of Minor Surgery. Review of, . . . . . . . . . . . . . . . 599
Ether, Sulphuric, . . . . . . . . . . . 294
Eunonymus, . . . . . . . . . . . . . . 470
Eupat-perf., . . 288, 291, 379, 388, 433, 528, 532
Eupion, . . . . . . . . . . . . . . . 439, 532
Eurich, Dr. C. Removal, . . . . . . 304
Evans, C. H., M. D. How to Cure Rhus Poisoning, . . . . . . . . . 509
Everly, W. E. Advice Wanted, . . . 118

PAGE
Fahnstock, J. C., M. D. Accidental Proving of Carbolic Acid, . . . 554
Felix-mas., . . . . . . . . . . . . . 295, 436
Ferri-carb., . . . . . . . . . . . . . . 338
Ferrum-aceticum, . . . . . . . . . . . 439
Ferrum-met., . . . . . . . . . . . . . 533
Fever, . . . . . . . . . . . . . . . 287
Few More Cases Cured with the High and Highest Potencies. A. J. Emmons, M. D., . . . . . . . . . 283
Fibroid Tumor of the Uterus. E. T. Adams, M. D., . . . . . . . . . . 241
Fincke, B., M. D. Potentiation Physiologically Proven, by Prof. Dr. Gustav Jaeger, . . 37, 119, 268, 314, 414, 450, 483, 535, 577
Clinical Cases, . . . . . . . . . . 98, 103
"Corrections" of Prof. Dr. Jaeger from Hahnemannian standpoint 119
Translator's Preface from the Translation of *The Organon*, . . 46
Fitch, John H., M. D. A Case of Insanity, . . . . . . . . . . . . . 343
Fluoric-acid, . . . . . . . . . 295, 379, 528
Formica, . . . . . . . . . . . . . 435, 533
Fowler, S. Mills, M. D. Removal, . . 63
Fracture of Leg, Compound. T. D. Stow, M. D., . . . . . . . . . . . 31
Free Trip to Chicago. Note, . . . . . 352
Fun for Doctors, . . . . . . . 64, 352, 512, 560
Furuncles, . . . . . . . . . . . . . 525

Gelsemium. Hydro-salpingitis, A Case Cured with. J. H. Allen, M.D., 244
Gelsem-nit. . . . 244, 379, 384, 439, 471, 528
Gettysburg-salt, . . . . . . . . . . 53, 490
Gettysburg Spring Water. . . . 433, 470, 533
Gilbert, Chas. B., M. D. Sciatica, . . 346
Note, . . . . . . . . . . . . . . . 255
Ginseng, . . . . 294, 384, 386, 470, 528 533
Glaucoma. A. G. Allan, M. D., . . . 3
Gleanings. F. H. Lutze, M. D., . 366, 474, 551
Grace Hospital, . . . . . . . . . . . 255, 304
Graph., . . . . . . . . . . . . . . . . 379
Guajacum, . . . . . . . . . . . 288, 388, 528
Guernsey, J. C., M. D. Homœopathic Medical Society of Penna., . . . 448
Guernsey, Wm. Jefferson, M. D. Repertory of Diphtheria, . . . . . . 222
Gummi-gutti, . . . . . . . . . . . . . 384
Gundlach. J. G., M. D. Sanicula—Clinical Verifications, . . . . . 158

Hahnemann Medical College of Phila. Introductory Address, . . . . . 563
Hahnemann, Statue of, . . . . . . . . 253
Hall, John. Vaccinium and Variolinum, . . . . . . . . . . . . 392, 510
Hamamelis virg., . . . . . . . 295, 439
Hamlet's Apostrophe to Man. Walter M. James, M. D.. . . . . . . . . 195
Haynes, J. R., M. D. An Open Letter, . . . . . . . . . . . . 188, 250
Heath, Alfred, M. D. British Medicinal Plants, . . . . . . . . . . . 394
Hellebore, . . . . . . . . . . . . . 492, 533
Hemorrhage following Abortion readily Controlled by the Indicated Remedy. J. A. Tomhagen, M.D., 246
Hepar-sulph., . . . . . . . . . . . . . 528
History of the Life of D. Hayes Agnew, M. D., LL. D. By J. Howe Adams, M. D. Review of, . . . 350
Hitchcock, Harlyn, M. D. In Memoriam, Samuel Swan, M. D., . . . 589
Holcombe, Dr. A. W., Located, . . . . 512
Holmes, Horace P., M. D. The Homœopathic Prescription over the Pathological One, . . . . . . . . 159
The Western Hahnemannian Club, 284
Homœopathic Aggravation. S. E. Chapman, M. D., . . . . . . . . . 427
Homœopathic Aggravation. Walter M. James, M. D., . . . . . . . . . 401
Homœopathic Medical College of Missouri, . . . . . . . . . . . . . 58, 250
Homœopathic Medical Society of Pennsylvania. J. C. Guernsey, . 448
Homœopathic Prescription over the Pathological One, The. Horace P. Holmes, M. D., . . . . . . . 158
Homœopathy in Animals. Dr. Reed, . 545
Homœopathy; World's Congress of, . 54
Homœopathy, Latest Crusade Against, Walter M. James, M. D., . . . . . 305
Homœopathy; What is Fittest in and Likely to Survive. Walter M. James, M. D., . . . . . . . . . 353
Horlick's Malted Milk, . . . . . . . . 256
Howard, C. C., M. D. Melancholia, . 115
Hydrastis-can., . . . . . . . . . . . 386
Hydrophobin, . . . . . . . . . . . 53, 288
Hydro-salpingitis. A Case Cured with Gelseminum. J. H. Allen, M.D., . 244
Hyoscyamus, . . . . . . . . . . . 288, 526
Hypericum-perfol., . . . . 379, 386, 439, 470

Illustration, An. Howard Crutcher, M. D., . . . . . . . . . . . . . 347
In Memoriam. Dr. Samuel Swan, 557, 589
Insanity, A Case of. John H. Fitch, M. D., . . . . . . . . . . . . . . 343
Intermittent Fever, Wells on, . . 450, 562
International Congress of Charities, Correction, and Philanthrophy, The, . . . . . . . . . . . . . . 255
International Hahnemannian Association, The, . . . . . . . . . 129, 255
International Hahnemannian Association, Proceedings of. Walter M. James, M. D, . . . . . . . . 129
Meeting of, 1892, . . . 2, 67, 129, 130, 197
The. J. B. S. King, M. D., . . . . 443
To the. Overton F. MacDonald, M. D. . . . . . . . . . . . . . . 148
International Medical Annual, The. By P. W. Williams, M. D. Review of, . . . . . . . . . . . . . 302
Introductory to the 46th Annual Course of Instruction in Hahnemann Medical College of Philadelphia. B. Frank Betts, M. D., 563
Iodide of Potassium, . . . . . . . . . 529
Iodine, 288, 295, 379, 386, 388, 389, 434, 436, 526
Ipecac, . . . . . . . . . . . . . . . . 529
Iris-vers., . . . . . . . . . . . . 295, 379, 533
Isopathy in the Dominant School. Walter M. James, M. D., . . . . 513

Jaeger, Prof. Dr. G. A Correction, . . 45
Potentiation Physiologically Proven. 37, 268, 314, 414, 450, 483, 535, 577
The Correction of. B. Fincke, M. D., . . . . . . . . . . . . . . 119
Work in America, . . . . . . . . 45
James, Prof. Bushrod W. Alaska, Review of, . . . . . . . . . . . . 60
James, Walter M., M. D. Address to Delinquent Subscribers, . . . . 561
The Delay in Issuing this Number. Editorial, . . . . . . . . . . . 1
Hamlet's Apostrophe to Man, . . 195
Homœopathic Aggravation. . . . 401
Isopathy in the Dominant School, 513
Lachesis and Lycopodium, . . . . 257
The Latest Crusade Against Homœopathy, . . . . . . . . . . 305
Neural Analysis, . . . . . . . . . 449
The Proceedings of the International Hahnamannian Association. . . . . . . . . . . . . 129
Pyrogen, . . . . . . . . . . . . . 401
Reforming the Materia Medica, . . 481
The Suppression of Symptoms, . . 467
What is the Best High Potency? . 65
What is Fittest in Homœopathy and Likely to Survive. . . . . . 353
Wells on Intermittent Fever, . 450, 562

PAGE
Jameson, R. E., M. D. Puerperal Convulsions, . . . . . . . . . . . . . 237
Jasper, . . . . . . . . . . . . . . . . . 492
Jump, Julia C., M. D. Apis—An Involuntary Proving, . . . . . . . . 396

Kali-bichr., . . . . . . . . . . . . . . 433
Kali-carbonicum, . . . . . . . . 295, 492, 533
Kali-hyd, . 51, 286, 379, 386, 434, 436, 489, 533
Kalmia-latifolia, . . . . . . . . . . 295, 379
Kaolin, . . . . . . . . . . . . 386, 434, 533
Kimball, Samuel A., M. D. A Case of Typhoid Pneumonia Cured with Hyos.dmm, . . . . . . . . . . . . 84
A Miscarriage Between the Fourth and Fifth Months, . . . . . . . . 234
King, J. B. S., M. D. The International Hahnemannian Association, 443
Klip, The. Note, . . . . . . . . . . 304
Krebs, Dr. F. H. Removal of, . . . . 560
Kreosote, . . . . . . . . 295, 388, 440, 474, 526

Lachesis and Lycopodium. Walter M. James, M. D., . . . . . . . . . . 257
Lachesis and Lycopodium; Comparison of Throat Symptoms of. E. V. Ross, M. D., . . . . . . . . . . 259
Lachesis, . . . . . . . . . 296, 434, 529, 534
Lachnanthes Tinctoria, . . . . . . . 76
Lactuca-viros, . . . . . . . . . 380, 529, 534
La Grippe, Child with, . . . . . . . . 208
Lamium-alb., . . . . . . . . . . . . 296
Lane, W. S., M. D. Threatened Miscarriage, . . . . . . . . . . . . 297
Latest Crusade Against Homœopathy, The. Walter M. James, M. D., 305
Laurocerasus, . . . . . . . . . . . . 529
Ledum-pal., . . . . . . . . . . . . . 295, 440
Ledyard, W. E., M. D. A Belladonna Case. Why not Mercurius? . . 164
Principles Involved in the Law of Cure, . . . . . . . . . . . . . 515
Surgical Cases (so-called) Cured Homœopathically, . . . . . . . . 17
Leonard, Wm. E., M. D. American Institute of Homœopathy, . . . 555
Libby Prison War Museum, The. Note, . . . . . . . . . . . . . . 351
Lilienthal, James, M. D. Appendicitis, 458
Lilium-tig., . . . . . . . . 295, 440, 471, 534
Linaria . . . . . . . . . . . . . . . . 440
Literary Note, . . . . . . . . . . . . . 303
Lithia-carb.5c., . . . . . . . . . . . . 51
Lochia, Allopathic Suppression of. D. C. McLaren, M. D., . . . . . . . . 247
Lowe, J. N., M. D. Carbuncle, . . . 23
Lutze, F. H., M. D. Gleanings, 366, 474, 551
Lycoperdon Bovista, . . . . . . . . . 78
Lycopodium in Diphtheria, . . . . . 166
Lycopodium, . . . . . . . . . . 166, 526, 534
Lycopodium and Lachesis; Comparison of Throat Symptoms of. E. V. Ross, M. D., . . . . . . . . 259

MacDonald, Overton F., M. D. To the International Hahnemannian Association. . . . . . . . . . . . 148
Macfarlan, Malcolm, M. D. Provings and Clinical Observations with High Potencies, . . . 50, 286, 375, 432, 488, 526
Skin Symptoms, . . . . . . . . . 50
Symptoms of Sleep, . . . . . . . 52
Mahony, Edward, M. D. Clinical Cases, with Remarks, . . . . . . 172
Surgical Cases, . . . . . . . . . . . 24
Malted Milk. Note, . . . . . . . . . 127
Martin, James T., M. D. The President's Address. . . . . . . . . . . 307
Mastitis, . . . . . . . . . . . . . . . 239
Materia Medica, Reforming the. Walter M. James, M. D., . . . . . . . 481
Materia Medica and Therapeutics, Practical Treatise on. By John V. Shoemaker, M. D. Review of, 445
Materia Medica, Therapeutics, and Repertory of the Digestive System, Compendium of, . . . . . 299
McLaren, D. C., M. D. Allopathic Suppression of Lochia, . . . . . 247
A Case of Acne, . . . . . . . . 92
McNeil, A., M. D. A Case of Auto-Isopathy, . . . . . . . . . . . . 372
Cases from Practice. Dr. Hesse, . 503
Cases Cured by Dr. Wassel, Keil, . 504
The Intermittent of Calcarea-carb., . . . . . . . . . . . . . . 345
Sycosis, . . . . . . . . . . . . . . 400
*Medical Advance, The.* Note, . . . . . 255
Medley, Jennie, M. D. Clinical Cases, 112
Melancholia. C. C. Howard, M. D., . 115
Mentha-pip., . . . . . . . . . . 384, 434, 471
Mephitis, . . . . . . . . . . . . . . . 529
Merc-corr., . . . . . . . . . . . . . 471, 490
Merc-iod., . . . . . . . . . . . . . . 296
Merc-prot-iod., . . . . . . . . . . . . 51, 471
Merc-viv., . . 296, 380, 391, 434, 436, 471, 535
Mezereum, . . . . . . . . . . . . . . . 296
Midway Sanitarium, The, . . . . . . . 128
Miscarriage Between the Fourth and Fifth Months, A. Samuel A. Kimball, M. D., . . . . . . . . . 234
Miscarriage, Threatened. W. S. Lane, M. D., . . . . . . . . . . . . . . 297
Missisquoi-water, . . . . . . . . . 296, 440
Missouri Institute of Homœopathy, The, . . . . . . . . . . . . . . 255
Modern Gynecology. By Charles H. Bushong, M. D. Review of. . . 350
Morgan, W. L., M. D. Clinical Cases, 141
Diseases of the Digestive Organs as the Primary Causes of Consumption and of Many Other Diseases, . . . . . . . . . . 130
Six Cases Treated Showing that Tuberculosis of the Joints Can be Cured by Medical Treatment, 137
Murder, A New Punishment for, . . . 398
Muriatic-acid, . . . . . . . . . 296, 440, 471
Myrica-cerif., . . . . . . . . . . . . 380
Myrrh, . . . . . . . . . . . . . . . . 492

Natrum-mur., . . . . 289, 290, 380, 384, 391
Neural Analysis. Walter M. James, M. D., . . . . . . . . . . . . . . 449
New York Homœopathic Medical College, . . . . . . . . . . . . . . 448
New York Homœopathic Union, Minutes of, . . . . . . . . . . . . . 594
New York State Hospital at Middletown. Note, . . . . . . . . . . 447
New York State Society, Resolutions of, . . . . . . . . . . . . . . 599
New York *World's* Greatest Year, The Note, . . . . . . . . . . . . . 128
Nichols, C. F., M. D. Belladonna as a Prophylactic . . . . . . . . . 347
Nitric-acid., . 296, 380, 388, 391, 434, 440, 471
Nitrum, . . . . . . . . . . 384, 388, 492, 534
Nosode Prescribing . . . . . . . . . . 580
Notes and Notices, 63, 127, 253, 303, 351, 400, 447, 512, 560, 600
Notes on the Newer Remedies Their Therapeutic Applications and Modes of Administration. By David Cerna, M. D. Review of, 252
Nux-vomica, 297, 380, 386, 391, 436, 440, 471, 529, 534

Onosmodium Virginianum. W. A. Yingling, M. D., . . . . . . . . . 358
Open Letter, An. J. R. Haynes, M.D., 188, 250
Open Letter, A Postscript. J. R. Haynes, M. D., . . . . . . . . . 250
Ophthalmic Diseases and Therapeutics. By A. B. Norton, M. D. Review of, . . . . . . . . . . . 61
Opium, . . . . . . . . . . . . . . . . 438
Our Meanest Crime. By John H. Clarke, M. D. Review of, . . . 61

PAGE
Ovaries, Repertory of the Symptoms of the. Cyrus M. Boger, M. D., 494
Ovi Gallinæ Pellicula, Ovi Membrani Vesicula Purkinge. Samuel Swan, M. D., . . . . . . . . . . . . 323
Oxalic Acid. Case of Hernia Cured by, 157
Oxalis, . . . . . . . . . . . 288, 380, 386, 492

Palladium, . . . . . . . . 286, 297, 435, 440
Patch, Dr. Note, . . . . . . . . . . . 256
Petrol, . . . . . . . . . 52, 375, 434, 440, 530
Phosphorus. . 381, 386, 435, 441, 492, 529, 535
Physician Himself, Book on the. Review of, . . . . . . . . . . . . . 302
Physostigma, . . . . . . . . . . . . . 490
Phytolacca-dec., 286, 289, 375, 381, 385, 391, 472, 530
Picric Acid, . . . . . . . . . . . . 76
Pix-liquid, . 375, 381, 387, 390, 392, 434, 535
Plantago-maj., . . . . . . 286, 289, 380, 472
Plantago-minor, 289, 375, 380, 389, 392, 435, 441, 472, 529, 534
Plumbum-acet., . . . . . . . . . 472
Pneumonia, . . . . . . . . . . . . 210, 218
Pneumonia, Typhoid, . . . . . 81, 84, 213
Polygonum, . . . . . . . . . . 436, 441, 472
Podophyl-pelt., . . . . . . . . . . . . 392
Polygonum, . . . . . . . . . 286, 389, 492
Pompili, G., M. D. Clinical Cases, . . 197
Potencies, A Few Cases Cured with the High and Highest. J. Emmons, M. D., . . . . . . . . . 283
Potency, What is the Best High. Walter M. James, M. D., . . . . . . . 65
Potentiation Physiologically Proven, by Prof. Dr. Gustav Jaeger. M.D. B. Fincke, M. D., . . 37, 268, 314, 414, 450. 483, 535, 577
Powel, Dr. Franklin. Removal of. . 63
Practical Treatise on Materia Medica and Therapeutics, with Especial Reference to the Clinical Application of Drugs, A. By John V. Shoemaker, A. M., M.D. Review of, . . . . . . . . . . . 445
President's Address, The. James T. Martin. M. D., . . . . . . . . . 307
Report of Committee on, . . . . . . 67
Principles Involved in the Law of Cure. W. E. Ledyard, M. D., . 515
Private Home of Dr. Milton J. Roberts, 194
Proceedings of the International Hahnemannian Association, The. Walter M. James, M. D., . . . . . 129
Professor Dr. G. Jaeger's Work in America, . . . . . . . . . . . . 45
Provings and Clinical Observations with High Potencies. Malcolm Macfarlan, M. D., . . 50, 286, 375 432 488, 526
Psorinum, Cases Cured by. I. Dever, M. D., . . . . . . . . . . . . . 88
Psorinum, . . 217, 292, 375, 380, 384, 387, 441, 472, 535
Psychopathia Sexualis. with Especial Reference to Contrary Sexual Instinct. By Dr. R. von Krafft-Ebing. Review of, . . . . . . . 478
Puerperal Convulsions. R. E. Jameson, M. D., . . . . . . . . . . . 237
Pulsatilla, . . . . . . . . . 381, 437, 534
Pyrogen, The Pathogenesis of. W. A Yingling, M. D., . . . . . . . . 444
Pyrogenum, Clinical Verifications of. G. W Sherbino, M D., . . . . 204
Pyrogenum (Pus from Septic Abscess). W. A. Yingling, M. D., . . . . 402
Pyrogenum, . . . . . . . . . . . . 210, 401

Quackenbush, Dr. A., has Succeeded Dr. Cook, . . . . . . . . . . . . . 560

Rabe, R. F., Jr. How to Cure Rhus Poisoning, . . . . . . . . . . . 445
Ranunculus-acris, . . . . 381, 387, 389, 392
Ranunculus-scel., . . . . . . . . . . . 381
Reed, W. L., M. D. Albuminuria, . . 69
Homœopathy in Animals, . . . . 545
Surgical Cases Treated Homœopathically, . . . . . . . . . . . . 9
Reminiscences and Criticisms. G. J. Waggoner, M. D., . . . . . . . . 332
Removal. Charles P. Beaman, M. D., 447
S. Mills Fowler. M. D. Note, . . . 63
Dr. E. P. Gregory, . . . . . . . . 63
Dr. F. H. Krebs, . . . . . . . . . 560
Dr. A. R. Morgan, . . . . . . . . . 63
Dr. Franklin Powel, . . . . . . . . 63
Dr. M. D. Satterlee, . . . . . . . . 127
Dr. Clarence M. Selfridge, . . . . 304
Dr. John Storer, . . . . . . . . . 512
Dr. J. W. Thomson. . . . . . . . . 447
Repertory, . . . . . . . . . . . . . 98
Repetition, The Question of. Howard Crutcher, M. D., . . . . . . . . 396
Report of Committee on President's Address, . . . . . . . . . . . . 67
Resignation of Dr. Bell, The. James B. Bell, M. D., . . . . . . . . . . 510
Resolutions of New York State Society, . . . . . . . . . . . . . . 599
Rhododendron-chrys., . . . . . . . . 390
Rhus Poisoning, How to Cure. C. H. Evans, M. D., . . . . . . . . . 509
R. F. Rabe, Jr., . . . . . . . . . . 445
Rhus-toxicodendron, . 52, 77, 287, 375, 381, 390, 392, 435, 445
Richardson, Wm. C., M. D., . . . . 63
Ringworm; Its Constitutional Nature and Cure. By J. Compton Burnett, M. D. Review of, . . . . . 446
Roger's Homœopathic Guide. By Dr. L. D. Rogers and Ida Wright Rogers, M. D. Review of, . . . 560
Ross, E. V., M. D. A Comparison of the Throat Symptoms of Lachesis and Lycopodium, . . . . . . 259
Rumex, . . . . . . . . . . . . . . . 530

Sabina, . . . . . . . . . . . . . . . . 79
Saccharum-lac., . . . . . . . . . . . 292
Salvia-off., . . . . . . . . . . . . . . 376
Sambucus-nig., . . . . . 287, 289, 376, 385, 392, 437
Sanguinaria, . . . . . . . . . . . . . 392
Sanicula—Clinical Verifications. J. G. Gundlach, M. D., . . . . . . . 158
Santonine, . . . . . . . . . . . . 381, 387
Sap-soda. . . . . . . . 52, 376, 381, 389, 535
Sarracenia-purp., . . . . 376, 382, 441, 535
Sassafras. . . . . . . . . . . . . . 390, 488
Satterlee, M. D., M. D. Has Removed, 127
Saunders, Mr. W. B., . . . . . . . . 64, 600
Scabies: Can it be Cured with the Simillimum? E. T. Adams, M.D., 144
Sciatica. Chas. B. Gilbert, M. D., . . 346
*Scientific American, The*, . . . . . . 128
Scientific Medicine—A Medical and Surgical Review. Thos. Wildes, M. D., . . . . . . . . . . . . . 261
Scutellaria-laterif., . . . . . . . . . . 292
Secale-corn., . . . . . . . . . . 290, 376, 382
Selfridge, Dr. Clarence M. Removal, 304
Senega, . . . . . . . . . . . . . . . 493
Sepia . . . . . . . . . . . 376, 382, 437, 492
Serpentaria, . . . . . . . . . . . . . 52, 376
Seward, S., M. D. Pathogenetic, or Toxic Effect of Tobacco, . . . . 202
Shall Hahnemann's *Chronic Diseases* be Reprinted? . . . . . . . . . 352
Sherbino, G. W., M. D. Clinical Verifications of Pyrogenum, . . . . 204
Siftings Saved. I. Dever, M. D., . . . 548
Silicea, . . . . . . . . . . . . . . . 385, 493
Skinner, Thomas, M. D. Curious Case of Double and Eccentric Vision—Cured by Two Doses of Sulphur[cm] (F. C.), . . . . . . . 364
Skin Symptoms. Malcolm Macfarlan, M. D., . . . . . . . . . . . . . 50

PAGE
Sleep, Symptoms of. M. Macfarlan, M. D., . . . . . . . . . . . . . . 52
Small, Edward Payson, M. D. The Editorial in the July Number, . . . 511
Special Diagnosis and Homœopathic Treatment of Diseases. By T. de Suzzi Verdi, M.D. Review of, 253
Spigelia, . . . . . . . . . . . . . . . . 382
Spongia, . . . . . . . . . . . . . . . 382, 530
Stannum, . . . . . . . . . . . . . . . . 441
Statue of Hahnemann, The, . . . . . 253
Stearn's Dose-Book. Review of, . . . 447
Steinrauf, William, M. D. Clinical Notes, . . . . . . . . . . . . . 348
Electro-Homœopathy, . . . . . . 399
Stover, Dr. John. Removed, . . . . . 512
Stow, T. D., M. D. Clinical Contribution, . . . . . . . . . . . . . . 217
Compound Fracture of Leg, . . . 31
Stramonium, . . . . . . . . . . . . . . 488
Strontia-carb., . . . . . . . . . 472, 488, 493
Sulphate of Morphia, . . . . . . . 382
Sulphur, C. M. (F. C.). Curious Case of Double and Eccentric Vision cured by two doses of. Thomas Skinner, M. D., . . . . . . . . . . 364
Sulphur, 364, 376, 382, 385, 434, 441, 488, 490, 527
Sulphuric acid, . . . . . . . . 435, 527, 530
Suppression of Symptoms, The. Walter M. James, M. D., . . . . . . . 467
Surgery, American Text-book of. Review of, . . . . . . . . . . . . 63
Surgery, Essentials of Minor. By Edward Martin, A. M., M. D. Review of, . . . . . . . . . . . . . 599
Surgical Cases (so-called) Cured Homœopathically. W. E. Ledyard, M. D., . . . . . . . . . . . . . . 17
Surgical Cases. Edward Mahony, M. D., . . . . . . . . . . . . . . 24
J. A. Tomhagen, M.D., . . . . . . . 34
Surgical Cases Treated Homœopathically. W. L. Reed, M. D., . . . 9
Surgical Operations. Two Successful Homœopathic. J. H. Allen, M. D., 14
Swan, Dr. Samuel. In Memoriam, 557, 589
Swan, Samuel, M. D. Ovi Gallinæ Pellicula Ovi Membrani Vescula Purkinge, . . . . . . . . . . . . 323
Sycosis. A. McNeil, M. D., . . . . . . 400
Sycotic Dysmenorrhœa. George H. Clark, M. D., . . . . . . . . . . . 94
Symphytum-off., 289, 376, 383, 387, 441, 473, 493
Symptoms of Sleep. Malcolm Macfarlan, M. D., . . . . . . . . . . . 52

Tabes Dorsalis, . . . . . . . . . . . . . 117
Taraxacum, . . . . . . . 377, 435, 468, 530
Tart-emet., . . . . . . . . . . . . . . . 383
Tellurium, . . . . . . . . . . . . . . . 377
Terebinth, . . 290, 376, 383, 385, 473, 488, 493
Therapeutics of Cholera. By P. C. Majumdar, M. D. Review of, . . 600
Text-Book of the Theory and Practice of Medicine. By William Pepper, M. D. Review of, . . . . . 251
Theridion, . . . . . . . . . . . 289, 435, 489
Thomson, Dr. J. W. has Removed, . . 447
Throat Symptoms of Lachesis and Lycopodium. Comparison of. E. V. Ross, M. D., . . . . . . . 259
Thuja, . . . . . . . . . . . . . . . 52, 77, 473
Tissue Remedies, Twelve, of Schussler. Review of, . . . . . . . 300
Tobacco, . . . . . . . . . . . . . . . . 531
Tobacco, Pathogenetic or Toxic Effect of. S. Seward, M. D., . . . . . 202
Tomhagen, J. A., M. D. Clinical Medicine, . . . . . . . . . . . . . . 219
Hemorrhage following Abortion Readily Controlled by the Indicated Remedy, . . . . . . . . . 246

PAGE
Tomhagen, J. A., M. D. Surgical Cases, . . . . . . . . . . . . . . . 34
Transactions of Forty-fifth Session of American Institute of Homœopathy. By Pemberton Dudley. Review of, . . . . . . . . . . . 480
Transactions of World's Congress of Homœopathy, . . . . . . . . . 511
Translator's Preface from the Translation of *The Organon*. B. Fincke, M. D., . . . . . . . . . 46
Trifolium-pretens, . . 377, 383, 390, 442, 473, 530
Triticum, . . . . . . . . . . . . . 383, 387
Trombidium, . . . . . . . . . . . . 289, 387
Tuberculosis of Bones and Joints. By N. Senn, M. D. Review of, . . 59
Tuberculosis of the Joints can be Cured by Medical Treatment, Six Cases Treated Showing that. W. L. Morgan, M. D., . . . . . . . 137
Tumor of the Neck Treated by the Use of the Knife followed by Death. F. S. Davis, M. D., . . . . . . . . 2
Tussilago, . . . . . . . . . . . . . 468, 473
Twelve Tissue Remedies of Schussler, The. By Wm. Boericke, M. D. Review of, . . . . . . . . . . . . 300
Typhoid Fever, Relapse from, . . . . 215
Typhoid Pneumonia, . . . . . . . . . . 213
B. Le Baron Baylies, M. D., . . . 81
Cured with Hyos.dmm, A Case of. Samuel A. Kimball, M. D., . . . 84

Uva-ursa, . . . . . . 377, 387, 442, 489, 531

Vaccination. Report of Committee on, . . . . . . . . . . . . . . . . 17
Vaccination, Value of, . . . . . . . 598
Vaccinium and Variolinum. John Hall, M. D., . . . . . . . . . 392, 510
Valerian, . . . . . . . . . . . . . . . . 489
Value of Vaccination, . . . . . . . . 598
Verbascum-thap., . . 389, 436, 468, 472, 493, 531
Vision; Double and Eccentric, Curious Case of, Cured by Two Doses of Sulphur. Thomas Skinner, M. D., . . . . . . . . . . . . . . 364

Waggoner, G. J., M. D. Reminiscences and Criticisms, . . . . . . . . . 332
Ward, John M'E., M. D. A case of Cuprum Poisoning. . . . . . . . 280
Wells on Intermittent Fever, . . 450, 562
Wesselhœft, W. P., M. D. A Case Cured with Argentum-nitricum, 110
Western Hahnemannian Club, The. Horace P. Holmes, M. D., . . 284
Whooping-Cough and Pneumonia Cured by Cuprum-metallicum, . 168
Wildes, Thomas, M. D. Scientific Medicine A Medical and Surgical Review, . . . . . . . . 261
Woorari, . . . . 289, 292, 377, 389, 392, 442
World's Congress of Homœopathic Physicians and Surgeons at Chicago. Note, . . . . . . . . . . 254
World's Congress of Homœopathy, A. Pemberton Dudley, M. D., Sec., 54
World's Congress Notes, . . . . . . . . 55
World's Congress Transactions. Pemberton Dudley, . . . . . . . . . 511

Yale Chair, The. Note, . . . . . . . 128
Yingling, W. A., M. D. Calendula-off., 337
Onosmodium Virginianum. . . . 358
Pyrogenum (Pus from Septic Abscess). . . . . . . . . . . . . . 402
The Pathogensis of Pyrogen, . . . 444

Zinc-met., . . . . . . . . . . . . . . . 392

Zinc-sulph., . . . . . . . 383, 392, 489, 531

Zingiber, . . . . . . . . . . . . . . . 472

Vol. XIII. JANUARY, 1893. No. 1.

### THE

### HOMŒOPATHIC PHYSICIAN,

502

A Monthly Journal of Homœopathic Materia Medica and Clinical Medicine.

*"He is a freeman whom truth makes free, and all are slaves beside."*
*"Seek the Truth: come whence it may, cost what it will."*

EDITED AND PUBLISHED BY

WALTER M. JAMES, M. D.

NOTICE.

***All articles for publication, books for review, business communications, etc., must be sent to Editor, 1125 Spruce Street, Philadelphia.***

***Subscribers will please notice that this Journal will be sent until arrears are paid and it is ordered discontinued.***

SURGEON GENL'S OFFICE. LIBRARY.

PHILADELPHIA:

